# Abnormal Expression of *DICER1* Leads to Dysregulation of Inflammatory Effectors in Human Synoviocytes

**DOI:** 10.1155/2019/6768504

**Published:** 2019-05-28

**Authors:** Congshan Jiang, Jing Xu, Wenhua Zhu, Yongsong Cai, Si Wang, Yuanxu Guo, Ke Xu, Manman Geng, Nazim Hussain, Yan Han, Fujun Zhang, Qilan Ning, Liesu Meng, Shemin Lu

**Affiliations:** ^1^Department of Biochemistry and Molecular Biology, School of Basic Medical Sciences, Xi'an Jiaotong University Health Science Center, No. 76 Yanta West Road, Xi'an, Shaanxi, China; ^2^Key Laboratory of Environment and Genes Related to Diseases (Xi'an Jiaotong University), Ministry of Education, China; ^3^Department of Orthopedics of the First Affiliated Hospital, Xi'an Jiaotong University Health Science Center, Xi'an, 710061, China; ^4^Department of Joint Surgery, Xi'an Hong Hui Hospital, Xi'an Jiaotong University Health Science Center, Xi'an, 710054, China; ^5^Center for Applied Molecular Biology (CAMB), University of the Punjab, Lahore Dy 681352, Pakistan

## Abstract

Dysregulation of multiple microRNAs widely takes place during rheumatoid arthritis (RA) and experimental arthritides. This study is performed to explore the possible mechanism underlying *DICER1* deficiency-mediated inflammation in human synoviocytes SW982. Firstly, RNAi of *DICER1* led to increased COX2, MMP3, and MMP13 protein production, while *DICER1* overexpression could reduce MMP13 expression. Secondly, the increase of IL-8 and decrease of TGF-*β*1 and TIMP1 were determined in the supernatant derived from *DICER1* siRNA-treated cells, while *DICER1* overexpression was found capable to reverse this effect. Ingenuity pathway analysis (IPA) software predicted that the *Dicer1* deficiency-induced dysregulated cytokines in synoviocytes could possibly lead to the inflammatory disorders in the synovial tissue. Moreover, *DICER1* deficiency could also reduce apoptosis, while *DICER1* overexpression was found to decrease the proliferation and enhance apoptosis. In addition, *DICER1* deficiency could lower the expression of multiple RA-related miRNAs such as miR-155. Meanwhile, *DICER1* overexpression could rescue their low expression levels. And then, gain or loss of miR-155 function could regulate the protein levels of MMP3 and MMP13. These results indicated that *DICER1* might play its role through regulating its downstream RA-related miRNAs. Our data demonstrated that *DICER1* deficiency could cause multiple proinflammatory events in human synoviocytes SW982. This mechanism study might provide the possible target molecule to modify the inflammatory destruction and overproliferation in synoviocytes.

## 1. Introduction

Mounting evidence demonstrated that dysregulation of microRNAs (miRNAs) widely takes place in rheumatoid arthritis (RA) and experimental arthritides. Many such molecules have been considered as RA-related miRNAs. miR-17-92 cluster (including miR-18a, 19a, 20a, and 92a) induced by TNF-*α* stimulation could activate primary rheumatoid arthritis synovial fibroblasts (RASFs) by increasing matrix metalloproteinase 1 (MMP-1), IL-6, IL-8, and monocyte chemoattractant protein 1 (MCP-1) and promoting chronic joint inflammation through positive feedback in NF-*κ*B signaling [1]. miR-19b was also able to exacerbate inflammation and induce NF-*κ*B signaling in primary RASF [2]. miR-26a could negatively regulate TLR3 signaling by targeting TLR3 itself in macrophages and ameliorate experimental arthritis in rats [3]. miR-152 was downregulated in arthritis rats, and its overexpression in FLS could reduce DNMT1 expression and the cell proliferation [4]. miR-30a-3p was found to target B cell-activating factor (BAFF) in RA fibroblast and hence negatively regulate the RA process [5]. miR-34a-3p was reported to target the X-linked inhibitor of apoptosis protein (XIAP), thereby mediating resistance of RASFs to apoptosis [6]. miR-124a could control cell proliferation of RA synoviocytes by targeting cyclic-dependent kinase 2 (CDK-2) and MCP-1 [7]. miR-155 was found to repress the levels of MMP3 in RASFs and hence might be involved in the modulation of the joint destruction process [8]. miR-221/222 and miR-323-3p were found increased in RASF, and miR-323-3p overexpression could promote Wnt/cadherin signaling pathways [9]. miR-346 regulated TNF-*α* production by targeting TTP, an RNA-binding protein which inhibited TNF-*α* synthesis in RA FLS [10].

Some of the above-mentioned RA-related miRNAs are widely considered to play a protective role and hence further studied as promising targets for arthritis therapy. For example, the administration of miR-146a duplex was found to prevent bone destruction during collagen-induced arthritis of mice [11]. Intra-articular injection of miR-15a duplex could upregulate caspase 3 and downregulate BCL-2 to induce apoptosis in synovium of autoantibody-mediated arthritis in DBA/1J mice [12]. Combination of miR-29 and miR-140 transfection could protect chondrocytes from IL-1*β*-induced cell matrix degradation in immature mouse articular chondrocytes [13]. Intra-articular delivery of miR-140-5p/3p could ameliorate autoimmune arthritis. In addition, transfection of miR-140-5p/3p into synovial fibroblasts could enhance cell apoptosis, while decline the proliferation and migration abilities [14].

These multiple dysregulated miRNAs and their potential use for arthritis therapy aroused a great attention of the scientific world. However, it is difficult to understand the molecular mechanism of miRNA participation in RA pathogenesis due to their hundreds of putative target mRNAs. More importantly, the mechanism for the altered expression of multiple miRNAs was largely unknown. It is surmised that there is some kind of dominator regulatory genes which are responsible for at least many of these dysregulation of these miRNAs. As we know, several crucial players such as *DROSHA*, *DGCR8*, *DICER1*, *TRBP2*, *XPO5*, and *AGO2* participate in miRNA universal biogenesis. One study found that the anti-Su autoantibodies from rheumatic patients' sera were capable of immunoprecipitating DICER1 protein, indicating its implication in rheumatic diseases [15]. However, the possible implication of *DICER1* in rheumatic disease has been long left unknown. Very recently, the downregulation of *DICER1* gene and several RA-related mature miRNA expressions was reported in synovial fibroblasts from RA patients, while *DICER1*-deficient mice with K/BxN serum-transfer arthritis also displayed an unbalanced miRNA profiles and an enhanced inflammatory response [16]. Hence, the crucial role of *DICER1* in RA was uncovered. However, the detailed mechanism is left unknown during maintenance of homeostasis during inflammatory responses. In our preliminary study, a reduced *DICER1* expression was also found in TNF-*α*- or IL-1*β*-stimulated human synoviocytes. This work further focuses on the possible mechanism for *DICER1* deficiency-mediated inflammation in human synoviocytes.

## 2. Materials and Methods

### 2.1. Cell Culture

Human synovial sarcoma cell line SW982 is highly recommended as a suitable human synoviocyte model for the study of RA therapy such as fluvastatin-induced apoptosis signaling [17] and hence was used in this study. The SW982 cell line was cultured in DMEM supplemented with 10% fetal bovine serum (FBS) and incubated at 37°C in humid conditions provided with 5% CO_2_. SW982 cells (2 × 10^5^ cells/ml) were plated into 6-well plates overnight before treated with various arthritis-related TLR ligands including 10 ng/ml PGN (TLR2 ligand), 10 *μ*g/ml poly I:C (PIC, TLR3 ligand), 10 ng/ml LPS (TLR4 ligand), and 3 *μ*g/ml imiquimod (TLR7 ligand) and inflammatory cytokines including 100 ng/ml recombinant human IFN-*γ* (PeproTech, USA), 10 ng/ml recombinant human IL-4 (PeproTech, USA), 10 ng/ml recombinant human TNF-*α* (PeproTech, USA), and 10 ng/ml recombinant human IL-1*β* (PeproTech, USA) for various time points. The cells were then harvested for RNA or protein isolation.

### 2.2. siRNAs and Plasmid Vectors

Human *DICER1* siRNA product was designed and synthesized by GenePharma Company (China), and the sequence information was described in Table 1. Ten nanomolar individual siRNA or various combinations of siRNA sets were used for cell transfection in 6-well plates. RNA and protein samples were harvested at 48 hours after transfection. Five-microgram plasmid carrying human *DICER1* full-length CDS (pCAGGS-hs-Dicer1 from Phil Shap's Lab, Addgene plasmid #41584 [18]) was transfected into each well in a 6-well plate for *DICER1* gene overexpression, and the plasmid pCAGGS-MCS empty vector was used as a control. Vector map and characteristics were plotted and described in Fig.  S1. Sequencing was also validated before use. Lipofectamine 2000 (Invitrogen, USA) was used for transfection of siRNAs or plasmid vector. Plasmids for cell transfection were prepared by using the EZNA™ Endo-Free Plasmid Kit (Omega, USA).

### 2.3. Transfection of miRNA Mimic or Inhibitor

Gain or loss of miR-155 function was achieved by using transfection of 10 nM miR-155 mimic or inhibitor with Lipofectamine 2000 for 48 hours. SW982 cells (2 × 10^5^ cells/ml) were plated into 6-well plates overnight before transfection. miR-155 mimic, inhibitor, or its negative control (described in Table 2) was transfected into cells with Lipofectamine 2000 for gain and loss of miR-155 function. Scrambled miRNA molecules with none target in human genome were chosen to serve as a negative control. RNA and protein were harvested within same batch during each independent cell experiment.

### 2.4. Reverse Transcription-Quantitative Polymerase Chain Reaction

Relative expression of protein-coding genes and miRNAs was determined by reverse transcription-quantitative polymerase chain reaction (RT-qPCR). Briefly, total RNA was harvested in the TRIzol® reagent (Invitrogen, USA), isolated using the phenol-chloroform method. RNA integrity and quantity was determined using NanoDrop 2000. A total RNA of 5 *μ*g for the universal cDNA RT reaction was reverse transcribed with the oligo d(T) primer using the RevertAid™ First-Strand cDNA Synthesis Kit (Thermo, USA). miRNA cDNA synthesis was performed using the mir-x miRNA first-strand synthesis kit (Clontech, USA) from 5 *μ*g total RNA.

Real-time PCR was performed by using Agilent Mx3000P (USA) with FastStart Universal SYBR Green Master (Roche, USA) for quantification. The expression of mRNA and miRNA was normalized against endogenous control *GAPDH* and U6 snRNA, respectively. All data were analyzed by using the 2^-ΔΔCt^ (semiquantification) method. The mRNA level of *DICER1* and miRNA level of miR-18a-5p, miR-19a-3p, miR-19b-3p, miR-20a-5p, miR-92a-3p, miR-26a-5p, miR-30a-3p, miR-34a-3p, miR-124-3p, miR-140-5p, miR-140-3p, miR-146a-5p, miR-155-5p, miR-221-3p, miR-222-3p, miR-323a-3p, and miR-346 were detected using qPCR. Primers for mRNA and miRNA detection were purchased from GENEWIZ Company (detailed information was described in Table 2).

### 2.5. Western Blotting

After cell treatment or transfection, lysates were collected using RIPA solution containing protease inhibitor cocktail, followed with incubation on ice for 30 min and centrifugation at 12,000 ×g for 15 min at 4°C. Supernatant was collected, quantified using the BCA protein quantification kit (Thermo, USA), and denatured at 99°C for 5 min. Protein samples with equal amount of 30 *μ*g were separated by 10% SDS-PAGE, then transferred to the PVDF membrane, and incubated with blocking solution and later with the primary antibody of anti-DICER1 (Abcam, USA), TLR3 (Biosen, China), COX2 (Abcam, USA), MMP3 (Abcam, USA), MMP8 (Proteintech, USA), MMP13 (Abcam, USA), and GAPDH (Proteintech, USA), respectively, at 4°C overnight. The membranes were washed with TBST, incubated with the secondary antibody (labeled with HRP) at room temperature for 1 h, and washed again before developed by using the SuperSignal® ECL West Pico kit (Thermo Scientific, USA) and captured by using the Syngene Image system, and the specific bands were scanned for density using Genesys softwares. The intensity of specific binding bands was calculated against the endogenous control (GAPDH), and data were showed as fold change against the control.

### 2.6. Cytokine Profiling Assay

To detect the secreted cytokine expression in cell supernatant, cytokine array was performed using RayBio® C-Series human cytokine antibody array C5. This array detects 80 human cytokines in conditioned cell culture media, as described in Table 3. Both the array detection and analysis were performed by RayBiotech Company. Briefly, cells (2 × 10^5^ cells/ml) in 6-well plates were transfected with *DICER1* siRNA for 4 h; then, the medium was changed into DMEM containing 0.2% FBS in total of 1.5 ml and incubated for next 48 h. The medium was collected and centrifuged at 2000 rpm for 10 min at 4°C, and the supernatant was stored at -80°C before assay. The raw intensity of dot immunoblot signal from array membrane incubated with the supernatant sample paired with a negative control was captured and showed in Results. The data was normalized using the internal loading control, and the data were further analyzed as signal fold change against the negative control group. Representative differentially expressed cytokines with fold change above ±2 including human IL-8, TGF-*β* and TIMP-1 were further confirmed using enzyme-linked immunosorbent assay (ELISA, kit purchased from Excell Biotech, China) according to the manufacturer's instruction.

### 2.7. Ingenuity Pathway Analysis

The list of all dysregulated cytokines (fold change beyond ±1.2) from this cytokine array was uploaded for bioinformatic calculation using the Ingenuity pathway analysis (IPA) software. The diseases and biofunctions related to these dysregulated cytokines during *Dicer1* deficiency were predicted according to the Ingenuity knowledge database (filtered by experimentally observed reference).

### 2.8. Cell Apoptosis and Proliferation

Flow cytometry was performed using Guava easyCyte 6 (Millipore, USA). Apoptosis was detected by using double staining of Annexin V-FITC and PI (7Sea Pharmatech, China). Mean fluorescence intensity of Annexin V-FITC staining was plotted with histogram, and double staining was displayed using scatter plots to discriminate the early, mid-late phase of apoptosis. Cell proliferation was detected using cell counting kit-8 (CCK-8) assay. Fold change of the optical density (OD) value from three independent cell experiments was used for data analysis.

### 2.9. Statistical Analysis

The experimental data were presented as mean ± standard error of the mean (SEM). The statistically significant difference between the experimental treatment group and the control group was calculated using SPSS 13.0 and plotted using GraphPad Prism. Differences between groups were analyzed by using the Mann-Whitney test. *P* < 0.05 was considered to be statistically significant.

## 3. Results

**Figure 1:**
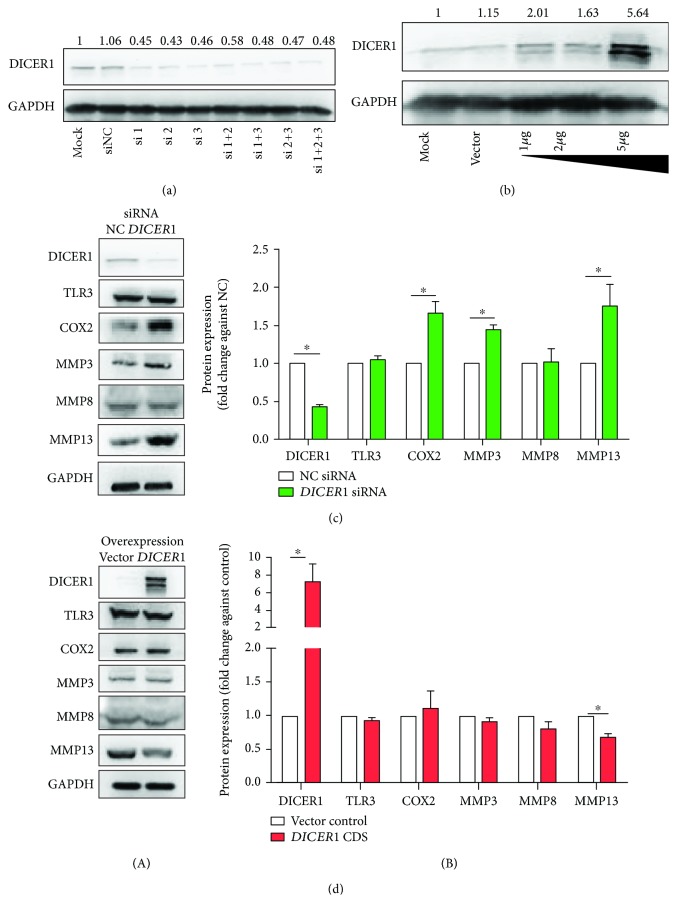
Altered intracellular protein expression caused by gain or loss of DICER1 function for 48 h in human synoviocytes SW982. (a) Representative Western blotting image of DICER1 expression after transfected with 10 nM DICER1 siRNAs 1, 2, and 3 or multiple combinations. (b) Representative Western blotting image of DICER1 expression after transfected with 1, 2, and 5 *μ*g overexpression vector per well in 6-well plates. (c) Western blotting results of DICER1, TLR3, COX2, MMP3, MMP8, MMP13, and GAPDH protein expression after DICER1 RNAi. (d) Western blotting results of DICER1, TLR3, COX2, MMP3, MMP8, MMP13, and GAPDH protein expression after *DICER1* overexpression. (A) Representative images; (B) quantitative results. Bar: mean ± SEM from three independent cell experiments, ^∗^ *P* < 0.05 using the Mann-Whitney test.

Responding to RA-related cytokine stimulation such as IL-1*β* and TNF-*α* rather than other cytokines (IFN-*γ* and IL-4) or TLR-activating ligands (TLR2, TLR3, TLR4, and TLR7 ligands), *DICER1* gene expression is significantly reduced at both the mRNA and protein levels in synoviocytes (Fig.  S2). Loss and gain of *DICER1* function were established in synoviocytes with *DICER1* knockdown (KD) or overexpression. KD efficiency of *DICER1* was verified using Western blotting methods. And the data showed that *DICER1* protein expression was successfully knocked down using siRNA molecules. To avoid the off-target effect, the combination of these three siRNAs was used in this study (Figure 1(a)). The DICER1 protein expression level in the cell transfected with the *DICER1* overexpression vector displayed a dose-dependent manner. It was demonstrated that 5 *μ*g vector per well in 6-well plates for *DICER1* overexpression is the optimal transfection condition (Figure 1(b)).

To explore altered intracellular protein expression caused by gain or loss of *DICER1* function, TLR3 (an RA-related important pattern recognition receptor), COX2 (a proliferation-related transcriptional factor), MMP3 (a proteinase for the extracellular matrix), and MMP8 and MMP13 (enzymes for type I and II collagen, respectively) were detected in synoviocytes with *DICER1* KD or overexpression for 48 h. The result showed that the expression of COX2, MMP3, and MMP13 was upregulated in the *DICER1* KD group, while MMP13 declined when *DICER1* was overexpressed (Figures 1(c) and 1(d)). These data suggest that loss of *DICER1* function might play a crucial role in the inflammation process of synoviocytes.

**Figure 2:**
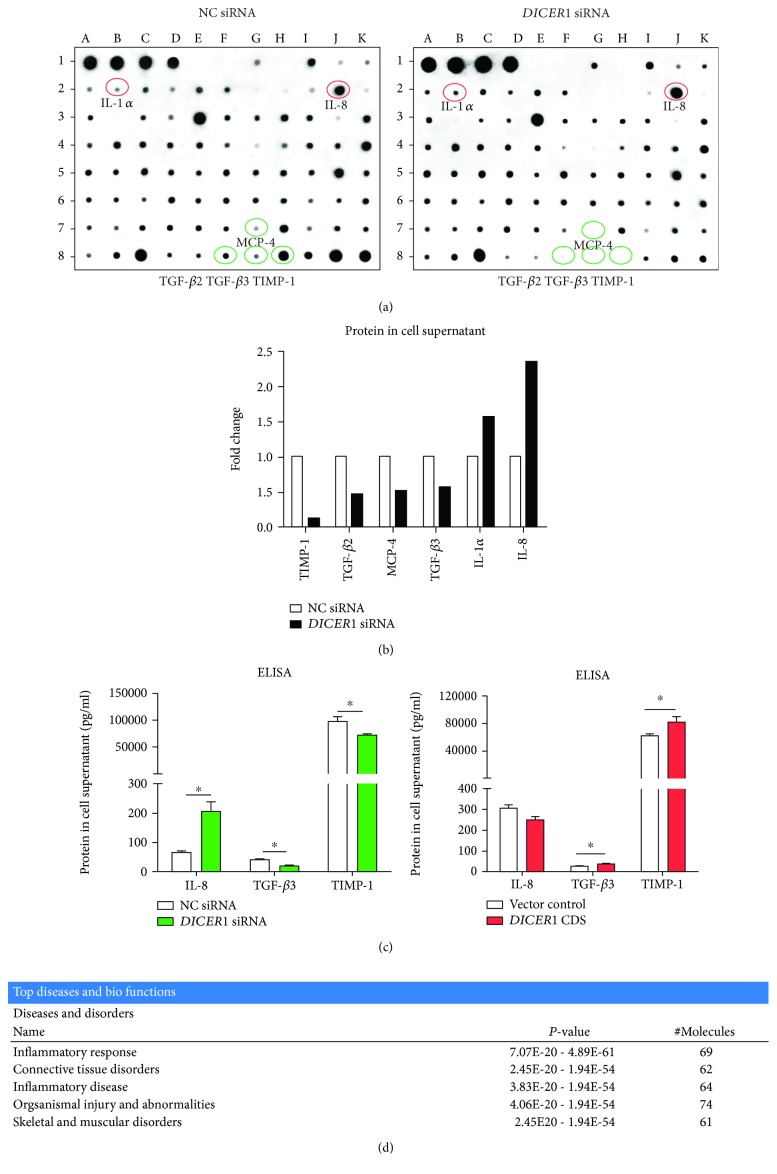
Altered production of cytokines and chemokines caused by gain or loss of *DICER1* function for 48 h in human synoviocytes SW982. (a) Image result of cytokine array detecting conditioned medium from cells transfected with *DICER1* or NC siRNA for 48 h. Two membranes were incubated with SW982 cell supernatant transfected by either negative control or *DICER1* siRNA for 48 h. (b) Fold change of cytokine expression in conditioned medium of the cell treated by *DICER1* siRNA for 48 h was detected by using cytokine array (quantitative result of (a)). Cytokines with fold change beyond ±1.5 were shown. (c) ELISA assay result for IL-8, TGF-*β*1, and TIMP1 cytokine expression in supernatant of SW982 cells with *DICER1* KD or overexpression. Bar: mean ± SEM from three independent transfection experiments. Triplicates were used for each transfection experiment, and duplicates were used in ELISA assay. ^∗^ *P* < 0.05 using the Mann-Whitney test. (d) IPA predicted top 5 diseases and biofunctions related to *Dicer1* deficiency-induced altered production of cytokines and chemokines.

Compared with a negative control, 6 out of 80 detected cytokines displayed obvious signal change on the membrane incubated with conditioned medium of SW982 cells after *DICER1* KD for 48 h (Figure 2(a)). Quantitative data indicated that the expression fold change of IL-1*α*, IL-8, TIMP-1, TGF-*β*2, TGF-*β*3, and MCP-4 was beyond ±1.5 (Figure 2(b)). In particular, the expression fold change of IL-8, TGF*β*2, and TIMP-1 was beyond ±2. To confirm the cytokine array data, the secreted cytokines in culture medium were further detected by using ELISA assay in three independent cell experiments. The data showed that in the *DICER1* KD group, IL-8 expression significantly increased, while TGF-*β*1 and TIMP1 declined. However, when *DICER1* was overexpressed, TGF*β*1 and TIMP1 increased significantly (Figure 2(c)). These data demonstrated that *DICER1* deficiency could lead to increased IL-8 and decreased TGF*β*1 and TIMP1 secretion. Rescue of *DICER1* function could also reverse these effects. Besides, according to the bioinformatic calculation using the Ingenuity pathway analysis (IPA) software (Figure 2(d)), the *Dicer1* deficiency-induced dysregulated cytokines were predicted to be strongly implicated in several diseases and biofunctions including inflammatory responses, connective tissue disorders, inflammatory diseases, organismal injury and abnormalities, and skeletal and muscular disorders. This hypothesis-free analysis further suggested that Dicer1 deficiency in synoviocytes could possibly lead to inflammatory disorders in the synovial tissue.

**Figure 3:**
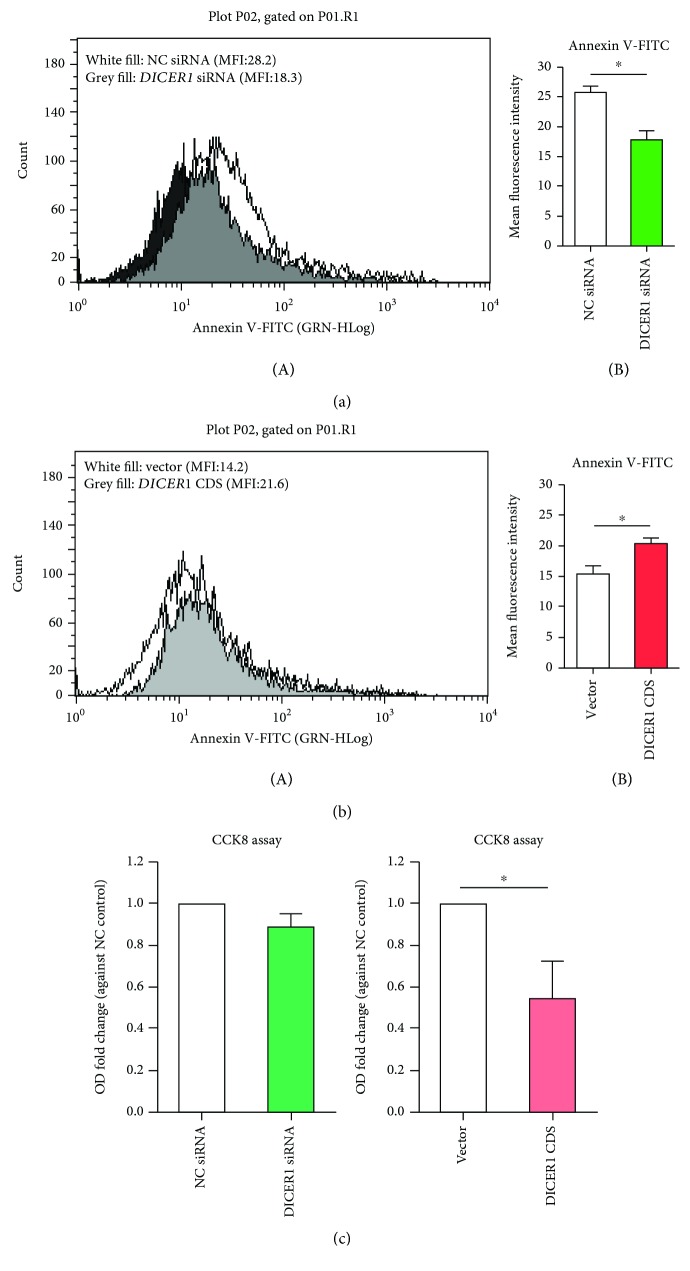
Altered apoptosis and proliferation caused by gain or loss of *DICER1* function for 48 h in human synoviocytes SW982. (a) Flow cytometry results of Annexin V-FITC staining after *DICER1* siRNA transfection for 48 h. (A) Representative histogram; (B) quantitative analysis of mean fluorescence intensity. (b) Flow cytometry results of Annexin V-FITC staining after DICER1 was overexpressed for 48 h. (A) Representative histogram; (B) quantitative analysis of mean fluorescence intensity. (c) Cell counting kit-8 assay for cell proliferation detection. Bar: mean ± SEM from three independent transfection experiments, and triplicates were used for each transfection experiment. ^∗^ *P* < 0.05 using the Mann-Whitney test.

Cell function assay such as proliferation and apoptosis was assessed in the *DICER1* KD or overexpression group. Mean fluorescence intensity (MFI) of Annexin V-FITC staining in SW982 significantly declined after *DICER1* KD; however, the MFI of Annexin V-FITC staining significantly increased when *DICER1* was overexpressed (Figures 3(a) and 3(b)). From the scatter plot of Annexin V-FITC and PI double staining, we could also found out that the apoptotic cell ratio (especially the early apoptosis phase) significantly declined in the *DICER1* KD group but increased in the overexpression group (Fig.  S3). Cell proliferation assay detected using cell counting kit-8 assay indicated that there is no change in the *DICER1* KD group, while after *DICER1* overexpression, cell proliferation becomes significantly slower (Figure 3(c)). These data suggest that *DICER1* deficiency causes resistance of apoptosis while its rescue could lead to more apoptotic cells and less proliferation.

**Figure 4:**
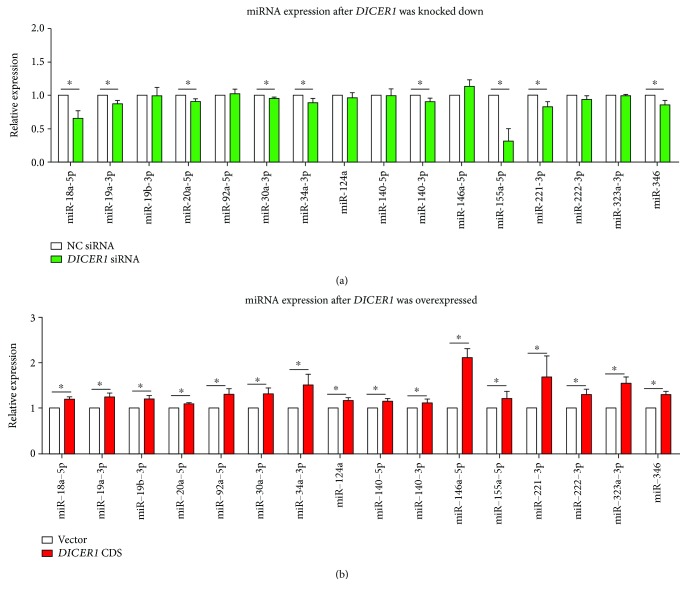
Altered expression of arthritis-related miRNAs caused by gain or loss of *DICER1* function for 48 h in SW982 human synoviocytes. (a, b) RT-qPCR result of 15 RA-related miRNAs in the *DICER1* knockdown (a) or overexpression (b) groups. Bar: mean ± SEM from three independent cell experiments, and triplicates were used for each cell transfection experiment. ^∗^ *P* < 0.05 using the Mann-Whitney test.

**Figure 5:**
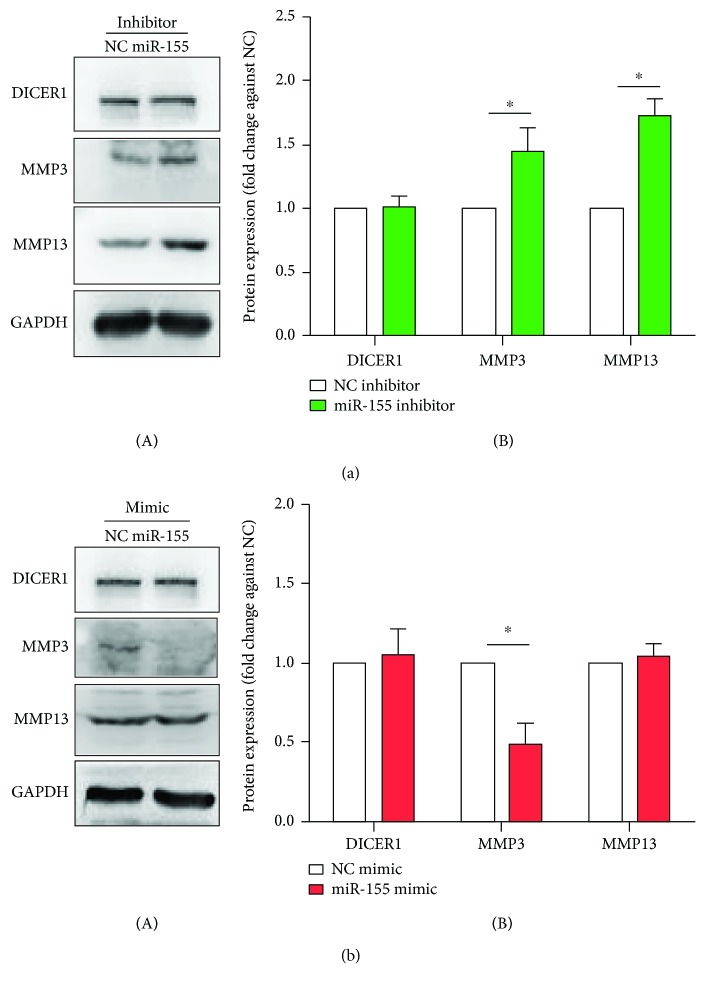
Altered expression of MMP3 and MMP13 caused by gain or loss of miR-155 function for 48 h in human synoviocytes SW982. (a) Western blotting result of DICER1, MMP3, and MMP13 in 10 nM miR-155 inhibitor-treated cells. (A) Representative images; (B) quantitative results. (b) Western blotting result of DICER1, MMP3, and MMP13 in 10 nM miR-155 mimic-treated cells. (A) Representative images; (B) quantitative results. Bar: mean ± SEM from three independent cell experiments, and triplicates were used for each cell transfection experiment. ^∗^ *P* < 0.05 using the Mann-Whitney test.

DICER1 as one of the crucial endoribonuclease in miRNA universal biogenesis, its altered function might lead to the dysregulation of downstream miRNAs. We detected multiple reported RA-related miRNAs including miR-18a-5p, miR-19a-3p, miR-18b-3p, miR-20a-5p, miR-92a-5p, miR-30a-3p, miR-34a-3p, miR-124a, miR-140a-5p, miR-140-3p, miR-146a-5p, miR-155a-5p, miR-221-3p, miR-222-3p, miR-323a-3p, and miR-346. The results showed that the expression of most of these mature miRNAs was reduced in the *DICER1* KD group, while all of these miRNAs increased after *DICER1* was overexpressed (Figure 4). Gain or loss of function from individual arthritis-related miRNAs such as miR-155 could also lead to similar expression regulation of proinflammatory proteins including MMP3 and MMP13 (Figure 5), which suggested that partial function of *DICER1* could be achieved through its downstream miRNAs.

## 4. Discussion

A reduced *DICER1* gene expression was found in synovial tissues from RA patients and TNF-*α*- or IL-1*β*-stimulated human synoviocytes SW982. In our study, it was showed that Dicer1 RNAi led to induced COX2, MMP3, and MMP13 protein products, while *DICER1* overexpression could reduce MMP13 expression. The increase of IL-8 and decrease of TGF-*β*1 and TIMP1 were detected in the supernatant derived from *DICER1* siRNA-treated cells, while *DICER1* overexpression was found capable to reverse this effect. IPA software predicted that the *Dicer1* deficiency-induced dysregulated cytokines in synoviocytes could possibly lead to inflammatory disorders in the synovial tissue. In addition, *DICER1* deficiency also caused less apoptotic cells, while *DICER1* overexpression could decrease the proliferation and increase apoptosis. Eventually, our data demonstrated that *DICER1* deficiency could lead to multiple dysregulated RA-related miRNAs especially miR-155, and gain or loss of its function could lead to similar molecular changes, indicating that *DICER1* might play its role through regulating its downstream RA-related miRNAs.

Consistent with the previous study [16], we believe that *DICER1* deficiency plays a crucial role in RA pathogenesis. Although our work found two important RA-related cytokines TNF-*α* and IL-1*β* as upstream regulators for *DICER1* gene expression, we did not address the detailed mechanism for *DICER1* upstream regulation. One ChIP-based study indicated that survivin could bind to the *DICER1* promoter region and survivin inhibition could lead to more than 5-fold change increase of *DICER1* expression. In leukocyte of 144 female RA patients, smoking as a risk factor was related to increased survivin transcription and hence decreased *DICER1* associated with low production of a mass of miRNAs [19]. Together with our work, these reports provide some important clues for the potential role of abnormal *DICER1* expression in pathogenesis of RA.

Apoptosis resistance, overproduction of destructive proteinases (matrix metalloproteinases and aggrecanases), and inflammatory cytokines and chemokines (IL-1*β*, IL-6, IL-8, and TNF-*α*) have been well described in human primary RA synoviocytes and widely recognized as potential targets for arthritis therapy [20]. TGF-*β*, however, is a very complicated cytokine during inflammation events. TGF-*β* plays a very active role in Th2-mediated immune response and tissue remodeling, e.g. it could upregulate IL-13 synthesis through GATA-3 expression in the T lymphocytes of patients with systemic sclerosis [21], while TGF-*β* signaling defect is linked to methotrexate resistance in rheumatoid arthritis [22]. Our result showed that *DICER1* RNAi led to less apoptotic cells, induced COX2, MMP3, and MMP13 protein products, increase of IL-8 secretion, and decrease of TGF-*β*1 and TIMP1 (tissue inhibitor of MMPs) secretion. These are very exciting experimental support, consistently demonstrating the proinflammatory role of *DICER1* deficiency in human synoviocytes.

Our data demonstrated that *DICER1* reduction could cause low expression of its dependent downstream miRNAs such as miR-155. Previously, miR-155 as the first dysregulated miRNA found in synovial tissues from RA patients was considered as a potent protective miRNA during RA. miR-155 was found to repress the levels of matrix metalloproteinase 3 (MMP-3) in RASFs and hence might be involved in modulation of the joint destruction process [8]. And it was also found that miR-155 played a profibrotic role by positively modulating TGF-*β*1 expression in proximal tubule cells [23]. These studies support our data that dysregulated *DICER1* could regulate its downstream RA-related miRNAs especially miR-155 to display its role in controlling MMP molecules, cytokine secretion, and so on.

The results showed that miR-155 inhibition led to the increase of MMP3 and MMP13 protein expression while miR-155 overexpression reduced MMP3 but not MMP13. According to the TarBase (the database for archived experimentally validated miRNA and mRNA interaction) [24] and the target prediction algorithm such as the TargetScan database, the MMP3 and MMP13 are less likely to be the direct target of miR-155. Hence, the regulation from miR-155 to MMP3 and MMP13 should be indirect. We consider that the negative regulation of MMP3 and MMP13 using anti-miR-155 might come from its role in negatively regulating the inflammatory pathways. Many inflammation-related genes were validated as miR-155 targets, such as NF-kappa B p65 [25], myeloid differentiation primary response protein 88 (MYD88) [26], and TAK1-binding protein 2 (TAB2) [27]. Hence, it is not hard to understand the anti-inflammatory role of miR-155 as well as MMP3 and MMP13 suppression. Some of these targets are positive regulators during toll-like receptor or NF-kappa B signaling, while others are negative players. For instance, one of its target genes, suppressor of cytokine signaling 1 (SOCS1), was a negative regulator for TLR signaling [28]. Besides, miR-155 was also reported to help in stabilizing the TNF-*α* mRNA molecule [29]. In summary, this single miRNA might play a very complicated role during many aspects of the inflammation. Hence, the additive consequence of gain or loss function for miR-155 could be neutralized for certain downstream molecules. This might be the reason why the mimic of miR-155 does not lead to MMP13 reduction as we assumed.

Although a human synovial fibroblast cell line SW982 is used to replace primary cells due to sample constraints, our results will provide an important theoretical and experimental basis for molecular events in RA patients and primary cells.

## 5. Conclusion

As all above mentioned, this work investigated the proinflammatory events during *DICER1* deficiency and explored how they eventually contributed to the inflammation in human synoviocytes SW982. Our work not only reveals the molecular mechanism underlying dysregulated *DICER1* gene and multiple RA-related miRNAs but also provides the possible target molecule to modify the inflammatory destruction and overproliferation in human synoviocytes.

## Figures and Tables

**Table 1:** Human *DICER1* siRNA, miR-155 mimic/inhibitor product sequence information.

Name	Product	Sequence (from 5′ to 3′)	Modification
NC siRNA	siRNA	Sense UUCUCCGAACGUGUCACGUTT Antisense ACGUGACACGUUCGGAGAATT	—

*DICER1* siRNA 1	siRNA	Sense GGACCAUUUACUGACAGAATT Antisense UUCUGUCAGUAAAUGGUCCTT	—

*DICER1* siRNA 2	siRNA	Sense GGCCAUUGGACACAUCAAUTT Antisense AUUGAUGUGUCCAAUGGCCTT	—

*DICER1* siRNA 3	siRNA	Sense CCUCCUGGUUAUGUAGUAATT Antisense UUACUACAUAACCAGGAGGTT	—

miRNA NC	Mimic	Sense UUCUCCGAACGUGUCACGUTT Antisense ACGUGACACGUUCGGAGAATT	—

miR-155-5p	Mimic	Sense UUAAUGCUAAUCGUGAUAGGGGU Antisense CCCUAUCACGAUUAGCAUUAAUU	—

miRNA NC	Inhibitor	CAGUACUUUUGUGUAGUACAA	2′Ome

miR-155-5p	Inhibitor	ACCCCUAUCACGAUUAGCAUUAA	2′Ome

NC: negative control.

**Table 2:** Primer information.

Gene symbol (full name)	Primer name	Ta (°C)	Sequences (from 5′ to 3′)
*DICER1* (Dicer1)	Sense	60	TGCAGTTCAGACAAGAGCAA
	Antisense		CAAAGCAGGGCTTTTCATTC
*GAPDH* (glyceraldehyde-3-phosphate dehydrogenase)	Sense	60	CACCCACTCCTCCACCTTTG
	Antisense		CCACCACCCTGTTGCTGTAG
*RNU6* (U6 small nuclear RNA)	Sense	60	CTCGCTTCGGCAGCACA
	Antisense		AACGCTTCACGAATTTGCGT
miR-18a-5p	Sense	60	TAAGGTGCATCTAGTGCAGATAG
miR-19a-3p	Sense	60	TGTGCAAATCTATGCAAAACTGA
miR-19b-3p	Sense	60	TGTGCAAATCCATGCAAAACTGA
miR-20a-5p	Sense	60	TAAAGTGCTTATAGTGCAGGTAG
miR-92a-3p	Sense	60	TATTGCACTTGTCCCGGCCTGT
miR-26a-5p	Sense	60	TTCAAGTAATCCAGGATAGGCT
miR-30a-3p	Sense	60	CTTTCAGTCGGATGTTTGCAGC
miR-34a-3p	Sense	60	CAATCAGCAAGTATACTGCCCT
miR-124-3p	Sense	60	TAAGGCACGCGGTGAATGCC
miR-140-5p	Sense	60	CAGTGGTTTTACCCTATGGTAG
miR-140-3p	Sense	60	TACCACAGGGTAGAACCACGG
miR-146a-5p	Sense	60	TGAGAACTGAATTCCATGGGTT
miR-155-5p	Sense	60	TTAATGCTAATCGTGATAGGGGT
miR-221-3p	Sense	60	AGCTACATTGTCTGCTGGGTTTC
miR-222-3p	Sense	60	AGCTACATCTGGCTACTGGGT
miR-323a-3p	Sense	60	CACATTACACGGTCGACCTCT
miR-346	Sense	60	TGTCTGCCCGCATGCCTGCCTCT

**Table 3:** Cytokine arrangement of RayBio® C-Series human cytokine antibody array C5 (detecting 80 human cytokines in conditioned cell culture media).

	A	B	C	D	E	F	G	H	I	J	K
1	POS	POS	POS	POS	NEG	NEG	ENA-78 (CXCL5)	G-CSF	GM-CSF	GRO a/b/g	GRO*α* (CXCL1)
2	I-309 (CCL1)	IL-1*α* (IL-1F1)	IL-1*β* (IL-1F2)	IL-2	IL-3	IL-4	IL-5	IL-6	IL-7	IL-8 (CXCL8)	IL-10
3	IL-12 p40/p70	IL-13	IL-15	IFN-*γ*	MCP-1 (CCL2)	MCP-2 (CCL8)	MCP-3 (CCL7)	M-CSF	MDC (CCL22)	MIG (CXCL9)	MIP-1*β* (CCL4)
4	MIP-1*δ*	RANTES (CCL5)	SCF	SDF-1*α*	TARC (CCL17)	TGF-*β*1	TNF-*α*	TNF-*β*	EGF	IGF-1	Angiogenin
5	OSM	TPO	VEGF-A	PDGF-BB	Leptin	BDNF	BLC (CXCL13)	Ck*β*8-1 (CCL23)	Eotaxin-1 (CCL11)	Eotaxin-2 (CCL24)	Eotaxin-3 (CCL26)
6	FGF-4	FGF-6	FGF-7 (KGF)	FGF-9	FLT-3 ligand	Fractalkine (CX3CL1)	GCP-2 (CXCL6)	GDNF	HGF	IGFBP-1	IGFBP-2
7	IGFBP-3	IGFBP-4	IL-16	IP-10 (CXCL10)	LIF	LIGHT (TNFSF14)	MCP-4 (CCL13)	MIF	MIP-3*α*	NAP-2 (CXCL7)	NT-3
8	NT-4	OPN (SPP1)	OPG (TNFRSF11)	PARC	PLGF	TGF-*β*2	TGF-*β*3	TIMP-1	TIMP-2	POS	POS

## Data Availability

Our additional evidence to support the conclusion of this study is described in supplementary data files. Besides, the original data used to support the findings of this study are available from the corresponding authors upon request.

## Abbreviations

CCK-8: Cell counting kit-8
ELISA: Enzyme-linked immunosorbent assay
FBS: Fetal bovine serum
IPA: Ingenuity pathway analysis
KD: Knockdown
miRNAs: MicroRNAs
MTX: Methotrexate
OD: Optical density
RT-qPCR: Reverse transcription-quantitative polymerase chain reaction
RA: Rheumatoid arthritis
RASF: Rheumatoid arthritis synovial fibroblast
RISC: RNA-induced silencing complex
SEM: Standard error of the mean.

## Supplementary Materials

Supplementary Materials Fig. S1: information of DICER1 overexpression plasmid. (A) Map of DICER1 overexpression plasmid (pCAGGS plasmid carrying full-length CDS). (B) Characteristics of plasmid carrying human DICER1 full-length CDS. Fig. S2: DICER1 mRNA and protein expression in TLR2,3,4,7 ligand or proinflammatory cytokine stimulated SW982 human synoviocytes. (A, B) Western blotting (A) and semiquantitative RT-PCR (B) of DICER1 expression in 10 ng/ml PGN (TLR2 ligand), 10 *μ*g/ml poly I:C (PIC, TLR3 ligand), 10 ng/ml LPS (TLR4 ligand), 3 *μ*g/ml imiquimod (TLR7 ligand), and inflammatory cytokines including 100 ng/ml recombinant human IFN-*γ*, 10 ng/ml recombinant human IL-4, 10 ng/ml recombinant human TNF-*α*, and 10 ng/ml recombinant human IL-1*β*-stimulated SW982 cells for 0, 6, 12, 24, and 48 h. Representative image from three independent cell experiments was shown. GAPDH was chosen as endogenous control for expression normalization during both the RNA and protein detection. Fig. S3: representative FACS imaging for altered apoptosis caused by gain or loss of DICER1 function for 48 h in SW982 human synoviocytes. (A) Altered apoptosis caused by loss of DICER1 function. (B) Altered apoptosis caused by gain of DICER1 function. Representative double staining of Annexin V-FITC and PI was displayed using scatter plots to discriminate the early, mid-late phase of apoptosis. Annexin V-FITC-positive cell percentage was analyzed for the apoptotic cell ratio. Mean ± SEM of data were analyzed from three independent transfection experiments; triplicates were used for each transfection experiment. ^∗^ *P* < 0.05 using the Mann-Whitney test.
