# Supplementary material for: Abnormal Expression of DICER1 Leads to Dysregulation of Inflammatory Effectors in Human Synoviocytes
Source: Mediators Inflamm. 2019 May 28;2019:6768504. doi: 10.1155/2019/6768504 (PMC6558604; doi:10.1155/2019/6768504)
Supplement: Supplementary Materials — Fig. S1: information of DICER1 overexpression plasmid. (A) Map of DICER1 overexpression plasmid (pCAGGS plasmid carrying full-length CDS). (B) Characteristics of plasmid carrying human DICER1 full-length CDS. Fig. S2: DICER1 mRNA and protein expression in TLR2,3,4,7 ligand or proinflammatory cytokine stimulated SW982 human synoviocytes. (A, B) Western blotting (A) and semiquantitative RT-PCR (B) of DICER1 expression in 10 ng/ml PGN (TLR2 ligand), 10 μg/ml poly I:C (PIC, TLR3 ligand), 10 ng/ml LPS (TLR4 ligand), 3 μg/ml imiquimod (TLR7 ligand), and inflammatory cytokines including 100 ng/ml recombinant human IFN-γ, 10 ng/ml recombinant human IL-4, 10 ng/ml recombinant human TNF-α, and 10 ng/ml recombinant human IL-1β-stimulated SW982 cells for 0, 6, 12, 24, and 48 h. Representative image from three independent cell experiments was shown. GAPDH was chosen as endogenous control for expression normalization during both the RNA and protein detection. Fig. S3: representative FACS imaging for altered apoptosis caused by gain or loss of DICER1 function for 48 h in SW982 human synoviocytes. (A) Altered apoptosis caused by loss of DICER1 function. (B) Altered apoptosis caused by gain of DICER1 function. Representative double staining of Annexin V-FITC and PI was displayed using scatter plots to discriminate the early, mid-late phase of apoptosis. Annexin V-FITC-positive cell percentage was analyzed for the apoptotic cell ratio. Mean ± SEM of data were analyzed from three independent transfection experiments; triplicates were used for each transfection experiment. ∗ P < 0.05 using the Mann-Whitney test. [file 6768504.f1.pdf]

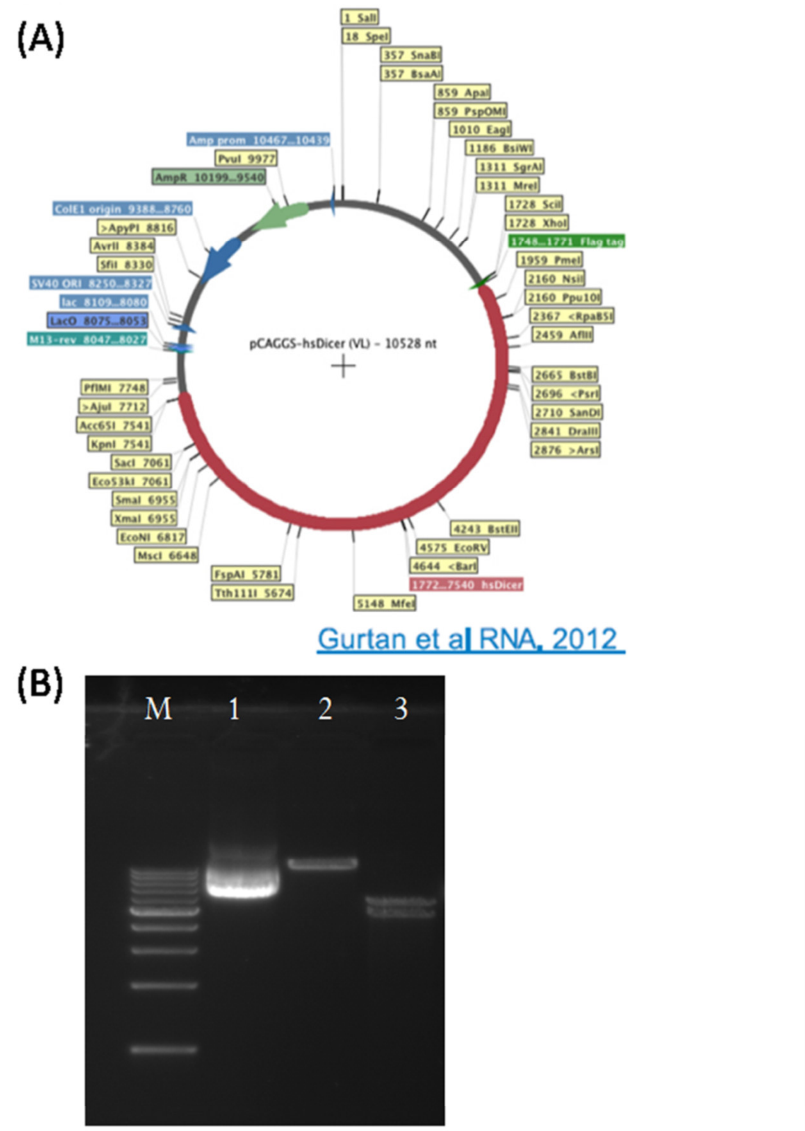

Marker: 1kb ladder
Lane 1-3: pCAGGS-human *DICER1* CDS
Lane 1: without digestion
Lane 2: Kpn I single digestion 10528bp
Lane 3: Kpn I, Xho I double digestion 5813, 4715bp

Fig.S1 Information of *DICER1* overexpression plasmid

(A) Map of *DICER1* overexpression plasmid (pCAGGS plasmid carrying full-length CDS)

(B) Characteristics of plasmid carrying human *DICER1* full-length CDS

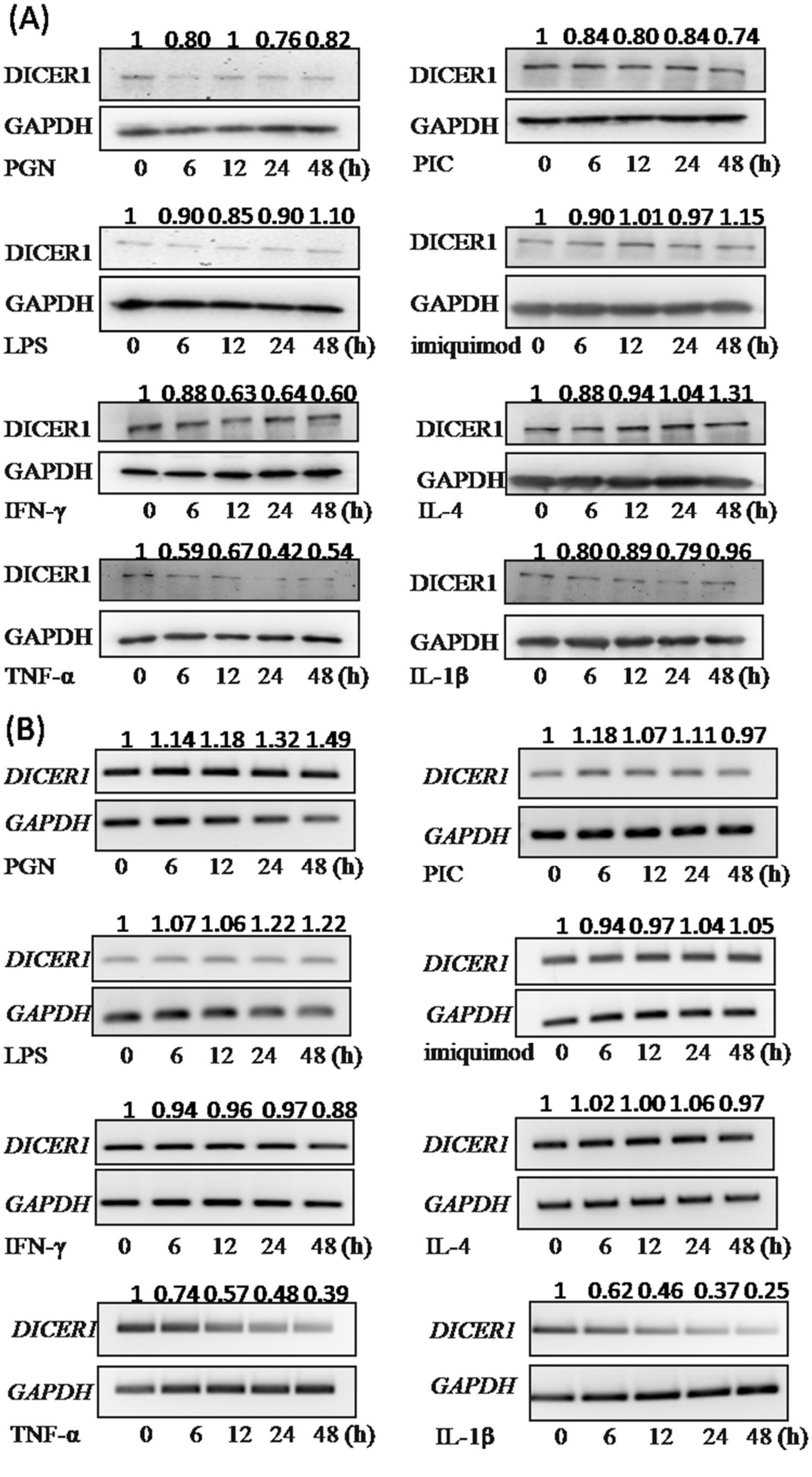

Fig.S2 *DICER1* mRNA and protein expression in TLR 2,3,4,7 ligand or

pro-inflammatory cytokine stimulated SW982 human synoviocytes.

(A, B) Western blotting (A) and semi-quantitative RT-PCR (B) of *DICER1* expression in 10ng/ml PGN (TLR2 ligand), 10μg/ml ploy I:C (PIC, TLR3 ligand), 10ng/ml LPS (TLR4 ligand), 3μg/ml immiquimod (TLR7 ligand) and inflammatory cytokines including 100ng/ml recombinant human IFN-γ, 10ng/ml recombinant human IL-4, 10ng/ml recombinant human TNF-α, 10ng/ml recombinant human IL-1β stimulated SW982 cells for 0, 6, 12, 24, 48h. Representative image from three independent cell experiments were shown. *GAPDH* was chosen as endogenous control for expression normalization during both the RNA and protein detection.

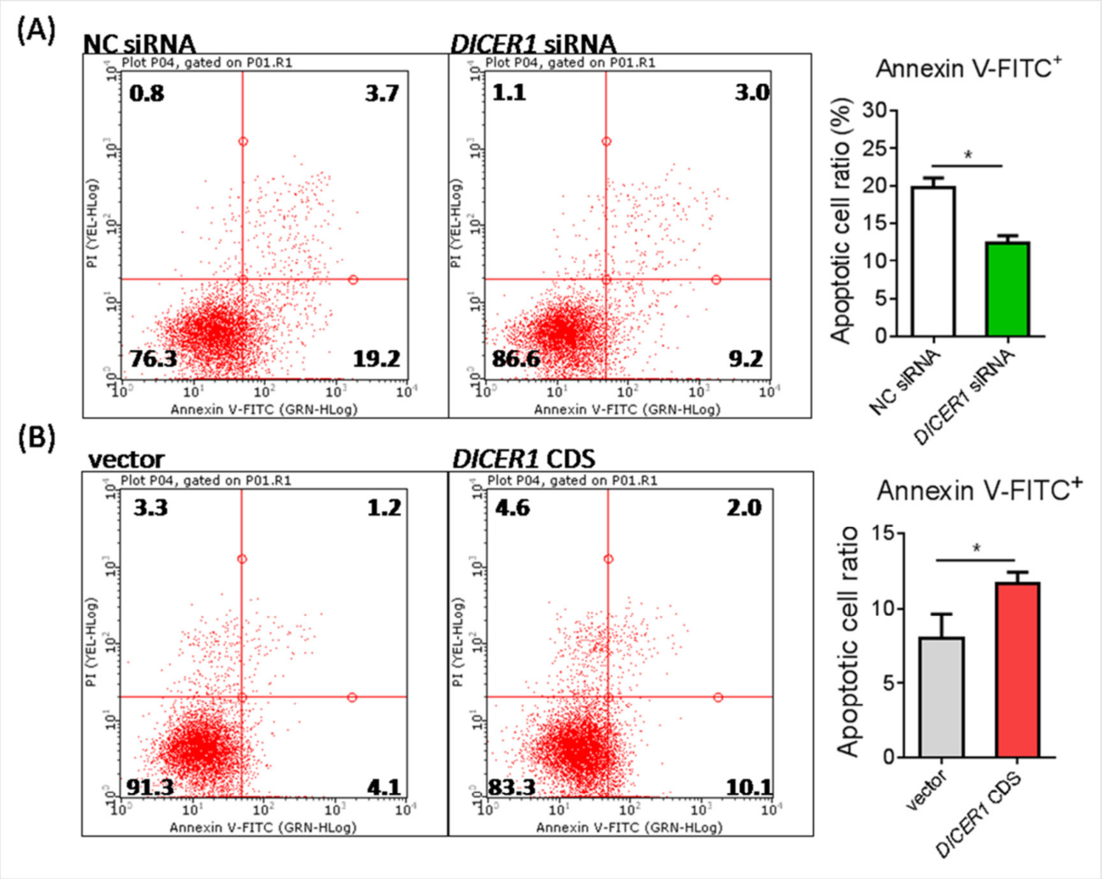

Fig.S3 Representative FACS imaging for altered apoptosis caused by gain or loss of *DICER1* function for 48h in SW982 human synoviocytes.

(A) Altered apoptosis caused by loss of *DICER1* function

(B) Altered apoptosis caused by gain of *DICER1* function

Representative double staining of Annexin V-FITC and PI was displayed using scatter plots to discriminate the early, mid-late phase of apoptosis. Annexin V-FITC positive cell percentage was analyzed for apoptotic cell ratio. Mean±SEM of Data were analyzed from three independent transfection experiments, triplicates were used for each transfection experiment. *: $p<0.05$ using Mann-Whitney test.
